# Editorial: Advances in mass spectrometry: transforming analytical chemistry in molecular and spatial biology, multimodal omics, and bioanalysis

**DOI:** 10.3389/fmolb.2026.1926838

**Published:** 2026-07-29

**Authors:** Andras Szeitz, Joana Pinto, Alex Pieters

**Affiliations:** 1 Genome Science and Technology Program, University of British Columbia, Vancouver, BC, Canada; 2 Life Sciences Institute, University of British Columbia, Vancouver, BC, Canada; 3 Applied Molecular Biosciences Unit (UCIBIO), Faculty of Pharmacy, University of Porto, Porto, Portugal; 4 Chemistry and Technology Network (REQUIMTE), Porto, Portugal; 5 School of Biomedical Engineering, University of British Columbia, Vancouver, BC, Canada

**Keywords:** analytical chemistry, mass spectrometry, metagenomics, multiomics, spatial biology

Mass spectrometry has emerged as a cornerstone technology in analytical chemistry, offering unrivalled sensitivity and specificity to decipher complex biological matrices and advance molecular science. Its wide range of applications has driven groundbreaking research in molecular biology (Guo et al., 2025), spatial omics (Liu et al., 2026), single-cell metabolomics (Petrova and Guler, 2025), and broader sphere of bioanalysis (Khalikova et al., 2024). As its scope of applications broadens with artificial intelligence and machine learning tools (Ebbels et al., 2023; Shenouda et al., 2026), mass spectrometry remains at the forefront of identifying, quantifying, and characterizing molecular entities over diverse biological disciplines. The current compilation, “Advances in Mass Spectrometry: Transforming Analytical Chemistry in Molecular and Spatial Biology, Multimodal Omics, and Bioanalysis”, presents 14 original research articles, two brief research reports, and two opinion papers from 141 authors, collectively showcasing recent advances in mass spectrometry techniques, microbial and metagenomic studies, spatial biology, and biotechnology. Together, these contributions highlight the evolving capabilities of bioanalytical mass spectrometry combined with analytical chemistry, emphasizing the technique’s adaptability and impact.

From the microbial and environmental systems perspective, Arini et al. presented a two-way integrative analysis of genomics and metabolomics data to characterize the marine bacterium BRA006 to discover its biosynthetic gene cluster (BGC) and bioactive compounds. Using Illumina and Oxford Nanopore sequencing combined with metabolomics and high-resolution mass spectrometry, they identified BRA006 as a new *Micromonospora* species and linked compound Brevianamide F to a BGC, which was previously incorrectly annotated. They demonstrated that integrated omics could correct and extend functional annotations of microbial genomes. Similarly, Islam et al. used an integrative approach in *Paracoccus denitrificans*, combining time-resolved targeted metabolomics with total proteomics to investigate how deletion of the *hnox* gene disrupts central carbon metabolism during growth phases. Their work highlighted the dysregulation of glycolysis, the pentose phosphate pathway, and energy metabolism and featured machine learning models to predict proteomic changes from metabolomic profiles They discussed the potential advantages and limitations of artificial intelligence in non-model microbial systems. Soares et al. investigated the microbial biotransformation of β-myrcene in *Pseudomonas* sp. M1, using adaptive laboratory evolution over 600 generations and employing comparative genomics, quantitative proteomics, and metabolite profiling. They found that two evolved lineages independently acquired mutations in FleQ, a master flagellar regulator, resulting in loss of motility but significantly higher final cell densities and enhanced metabolic flux through the myrcene pathway. Their work identified FleQ as a key adaptive target during β-myrcene-driven evolution and revealed various proteometabolic approaches to improve monoterpene bioconversion. Expanding on the high-resolution mass spectrometry implementation, Ayala-Ortiz et al. examined carbon cycling in palsa peat using multimodal metabolomics combined with ^13^C stable isotope-assisted metabolomics. Their molecule-level tracking showed that plant litter addition caused a short-term increase of CO_2_ emission fueled almost entirely by litter carbon, without substantially accelerating the decomposition of pre-existing peat organic matter. Their finding showed that litter inputs temporarily increased CO_2_ emissions, and peatland carbon stores remained relatively stable. Otto et al. developed a rapid, acid-hydrolysis-based method employing high-resolution mass spectrometry for global DNA methylation quantification using 5-methylcytosine and 6-methyladenine. They applied the method on the marine macroalga *Ulva mutabilis*, and the procedure offered a fast, sensitive, and cost-efficient alternative to sequencing techniques, providing global methylome information without the need for extensive bioinformatic analyses.

Several studies focused on biomarker discovery in human disease. Zhou et al. applied a non-targeted metabolomics approach with high-resolution mass spectrometry to investigate the mechanisms of icariin in the treatment of rheumatoid arthritis. They identified 31 potential biomarkers to diagnose metabolic disorders in rheumatoid arthritis, 23 of which were regulated by icariin, through pathways including the glutamate, arachidonic acid, pyruvate metabolism, the citrate cycle, and glycolysis, implicating oxidative stress and inflammation. Concomitantly, Masood et al. conducted a complementary investigation of rheumatoid arthritis using untargeted high-resolution mass spectrometry on 122 human plasma samples. They identified 300 dysregulated metabolites, ultimately annotating 60 endogenous metabolites with strong separation between rheumatoid arthritis patients and controls. They found major metabolic changes in the lipid, amino acid, and nucleotide metabolism pathways linked to cytokines and the MAPK pathway that supported plasma metabolomics as a phenotyping tool to find candidate biomarkers to diagnose rheumatoid arthritis. Wan et al. examined carotid atherosclerosis using metabolomics, spatial metabolomics, and single-cell transcriptomics with MALDI-MSI. They identified 74 differential metabolites between stable and unstable plaques of the patients, with arachidonic acid as the main inflammation-causing lipid upregulated in unstable plaques and enriched in 22 metabolic pathways. Spatial MALDI-MSI directly localized arachidonic acid with inflammatory macrophages, and single-cell analysis highlighted *ELOVL5* and *ALOX5* as over-expressed macrophage genes, offering mechanistic information on the linoleic acid–arachidonic acid–leukotriene D4 metabolic axis within atherosclerotic plaques. Dong et al. employed metabolomics to identify early biomarkers to detect acute intra-abdominal infection. They enrolled 30 patients and 20 healthy controls and analyzed their serum and urine samples with high-resolution mass spectrometry. They identified five serum biomarkers linked to fatty acid biosynthesis pathway, and two urinary biomarkers linked to catecholamine biosynthesis pathway. They proposed these candidates as diagnostic biomarkers for the early diagnosis of acute intra-abdominal infection. Zhang et al. developed and validated a rapid triple quadrupole mass spectrometric method for the simultaneous quantification of 11 antimicrobials in human plasma, achieving baseline separation of the components within 8 minutes. They validated the method according to regulatory guidelines, meeting the requirements for clinical therapeutic drug monitoring implementation. They successfully applied the procedure to measure plasma samples of pediatric patients receiving mono- or combination antibiotic therapy, building strategies for timely therapeutic drug monitoring in the clinic.

From the point of inflammation care, aging, and plant-based therapeutics, Xie et al. used untargeted metabolomics with high-resolution mass spectrometry to investigate *Cuscutae Semen* in a mouse model of depression-induced ovarian dysfunction. They identified ten differential metabolites from various pathways including arachidonic acid metabolism and steroid hormone biosynthesis, with seven target genes further validating their findings. The results supported *Cuscutae Semen* as a functional medical food with defined pharmacological mechanisms. Li et al. employed high-resolution mass spectrometry, non-targeted metabolomics, network pharmacology, and molecular dynamics to study Taohong Siwu Decoction in carbon tetrachloride-induced hepatic fibrosis. They identified 45 blood components, 148 differential metabolites, and a target network pointing to PI3K-Akt, MAPK, and estrogen signaling. They found ferulic acid derivatives as emerging key active compounds, with 3-hydroxy-4-methoxycinnamic acid showing stable binding to PPARG. Ying et al. took a transcriptomic approach to sarcopenia, integrating several public datasets to identify a three-gene mitochondrial signature, validating them *in vitro*, and achieving a high diagnostic area-under-the-curve (AUC) score. Their nomogram model emphasized the role of mitochondrial dysfunction in muscle aging and offered a potential clinical tool for early sarcopenia detection.

Working on clinical and respiratory applications, Gisler et al. explored real-time breath analysis using secondary electrospray ionization coupled with high-resolution mass spectrometry in 128 children (tobacco-exposed and non-exposed) and found that 71 breath features significantly correlated with the urinary oxidative stress marker 8-iso-PGF2α. They could not detect a strong correlation between predicted and measured values, but the study supported breath metabolomics as a promising non-invasive tool to indicate oxidative stress. Likewise, Shajari et al. monitored inflammatory bowel disease activity in 174 stool proteomics samples obtained by high-resolution mass spectrometry with data-independent analysis. A nine-peptide panel from five proteins, selected within a leakage-free nested cross-validation framework, achieved an outer-fold AUC of 0.93. More importantly, the panel retained diagnostic power (AUC 0.80) even in the calprotectin “gray zone,” where current non-invasive tests were unreliable.

From a bibliometric perspective, Wu et al. provided a broader context through the analysis of 1,188 publications on osteosarcoma metabolomics from 1995 to 2024. China leads in output, with growing interest in extracellular vesicles, immune metabolism, and single-cell metabolomics, reflecting a field-wide shift toward higher-resolution metabolic characterization and translational application.

Finally, Szeitz et al. hosted a two-part roundtable discussion where they brought together representatives from the mass spectrometry industry to highlight recent developments, research requirements, and regulatory guidelines on mass spectrometry instrumentation and technology. In the first part, the panelists explored current trends in mass spectrometry focusing on research applications and gaps, single-cell analysis, metabolomics, lipidomics, and multi-omics integration, while in the second part, they examined data management, bioinformatics, collaboration and industry needs. They sought to integrate mindsets within scientific research, inspire diverse perspectives, and foster scientific discourse with mass spectrometry transforming the landscape of medical science and molecular research.

Taken together, these studies showcase how metabolomics, integrated with genomics, proteomics, spatial imaging, and machine learning, is becoming central to mechanistic biology, clinical diagnostics, and therapeutic discovery through various biological systems.
